# Pei regimen: a therapeutic option in small cell lung cancer? A retrospective monoinstitutional analysis of 46 consecutive cases

**DOI:** 10.1186/s12967-015-0491-3

**Published:** 2015-04-24

**Authors:** Corrado Boni, Maria Pagano, Licia Baldi, Roberta Gnoni, Luca Braglia, Luisa Savoldi, Francesca Zanelli

**Affiliations:** Department of Oncology and Advanced Technologies, Oncology Unit, Azienda Ospedaliera S.Maria Nuova/IRCCS of Reggio Emilia, Viale Risorgimento, 80, 42123 Reggio Emilia, (Italy); Department Infrastructure Research and Statistics, Azienda Ospedaliera S.Maria Nuova/IRCCS of Reggio Emilia, Reggio Emilia, (Italy)

**Keywords:** Small cell lung cancer, Extended disease, Limited disease, Chemotherapy, Cisplatin, Etoposide, Ifosfamide

## Abstract

**Objectives:**

Combination chemotherapy is very active in small cell lung cancer (SCLC), although no improvement in overall survival (OS) has been done in the last 25 years, with Cisplatin-Etoposide (PE) still considered the world-wide standard, with an average median survival of about 7–8 months in patients with extended disease (ED).

In 1995, a randomized trial of the Hoosier Group in 171 ED patients showed a significant advantage in overall survival in patients treated with PEI (Cisplatin, Etoposide and Ifosfamide), compared to PE. Despite that, PEI regimen has not become a commonly used regimen in SCLC.

**Materials and methods:**

Here we present a retrospective analysis of 46 consecutive patients (30 males and 16 females) with SCLC that were treated at our Institution with PEI regimen: Cisplatin 20 mg/m2, Etoposide 75 mg/m2 and Ifosfamide 1200 mg/m2, day 1 to 4, every 3 weeks.

Patients received a total of 219 cycles of chemotherapy, with a mean of 4,7 cycles per patient. Median age was 63 (range 59–70); performance status (PS) was 0 in 29 patients (63%), 1 in 13 patients (28%) and 2 in 4 patients (9%).

**Results:**

In 19 limited disease (LD) patients partial response (PR) rate was 74%, and complete response (CR) was 16%. In 27 ED patients we observed 63% of PR and 26% of CR.

Median time to progression (TTP) was 15.2 months in LD and 7.1 months in ED with median overall survival (OS) of 28.2 and 11.8 months, respectively.

Toxicity was manageable, with a high dose intensity.

**Conclusions:**

PEI regimen, in our opinion, may be a possible therapeutic option, with high activity and an acceptable toxicity profile.

**Trial registration:**

ClinicalTrials.gov Identifier: NCT02324296.

**Institutional review board that approved the study:**

Institutional review board of Reggio Emilia, Azienda Ospedaliera S.Maria Nuova/IRCCS.

## Introduction

Neuroendocrine tumors account for approximately 20% of lung cancers; most of them (≈80%) are SCLC [1]. In 2012, about 34,000 new cases of SCLC have occurred in the United States, in the majority attributable to cigarette smoking [2].

SCLC tends to disseminate early in the course of its natural history and to grow quickly. Approximately 10% to 18% of patients present with brain metastases (BM) at the time of initial diagnosis, and in an additional 40% to 50% will develop BM some time during course of their disease [3].

Although the incidence of SCLC is decreased in recent years, it remains a therapeutic challenge, as survival in patients with limited disease has not changed markedly over the past 25 years, reaching approximately 20% to 25% at 5 years in the best published series of patients treated with a multimodality approach [4]

SCLC has a propensity for early hematogenous spread and for association with paraneoplastic syndromes and is characterized by its rapid doubling time, high growth fraction and high, but short-lasting, chemosensitivity.

Paraneoplastic syndromes are more frequently seen in patients with limited-stage SCLC. Than in those with extensive-stage disease, but their presence is not unequivocally prognostically favourable [5,6].

Chemotherapy is the cornerstone of treatment and Cisplatin-Etoposide (PE) is still considered the world-wide standard since over 25 years.

Approximately one-third of patients diagnosed with SCLC present with LD, with a median survival time of 15–20 months, compared to 7–8 months for patients with ED. An overall response to combination chemotherapy is achieved in 80-90% of LD patients and in 60-80% of patients with ED [7].

Also the complete response rate is influenced by the extension of the disease, and is significantly higher in patients with limited disease (30-50%) compared to patients with extended disease (15-30%) [8].

Since 90s, several studies have been conducted adding ifosfamide, an analogue of cyclophosphamide, to the PE combination, based on his single-agent activity and low toxicity [9].

The randomized trial of the Hoosier Oncology Group in 171 ED pts showed a significant advantage in overall survival in the arm treated with PEI, compared to PE [10–12]. Despite that, PEI has not become a commonly used regimen in SCLC, maybe because its less convenient schedule, requiring 4 consecutive days of chemotherapy. Relying on its efficacy and manageable toxicity, PEI was the reference schedule for the treatment of SCLC, in our centre since 15 years.

Primary objective of the present retrospective study is to evaluate the activity of PEI in the SCLC in terms of: response rate, time to progression and overall survival, in patient with LD or ED, treated in first line. Secondary objective were: the analysis of the dose intensity of chemotherapy and the evaluation of the tolerability in terms of toxicity and adverse events.

## Material and methods

Between December 1998 and December 2008, 46 consecutive patients, older than 18 years, performans status (PS) <2, with localized (LD) and extensive stage (ED) SCLC presenting to the Medical Oncology Unit of Reggio Emilia (Italy) were treated, in first line, with PEI regimen. All eligible patients had histologically or cytologically proven SCLC, with measurable disease defined by RECIST criteria, and received at least one cycle of chemotherapy. Patients with central nervous system (CNS) metastases were included in the study.

Pretreatment evaluation included physical examination, bronchoscopy, total body computed tomography (CT), and blood tests including a complete blood count, platelet count, serum creatinine and liver enzyme determination.

The study was approved by our local Ethics committee. All patients gave written informed consensus before starting chemotherapy with PEI regimen, and at the time of data analysis all living patients gave another written informed consent for the use of their personal data in the study analysis.

### Treatment regimen

The PEI regimen consisted of Cisplatin (P) 20 mg/m^2^ intravenously (IV) infused in 30 minute, on days 1 to 4 with pre hydration with 1000 ml of 5% dextrose and 2000 ml of 9% normal saline over 2 hours and mannitol-induced diuresis, Etoposide (E) 75 mg/m^2^ IV infused in 60 minute, on days 1 to 4 and Ifosfamide (I) 1200 mg/m^2^ IV infused in 60 minute, on days 1 to 4. Mesna was administered at the dose of 1200 mg total by IV bolus before the first dose of Ifosfamide and then after 4 and 8 hours at the same dose. Urinalysis was performed daily during the Ifosfamide treatment. Courses were repeated every 3 week for 4–6 cycles unless demonstration of disease progression or inacceptable toxicity.

All patients received intravenous 5-HT3 antagonists and dexamethasone as antiemetic prophylaxis, for the 4 days of treatment.

All drugs were reduced by 25% in case of G3 haematological toxicity. In case of febrile neutropenia or thrombocytopenia, requiring platelet transfusion, all drugs were reduced by 50% and, in such cases, no re-escalation was performed.

The use of colony stimulating factors were allowed but not included as part of the standard treatment regimen. Drug administration was postponed by 1 week if there was no full haematological recovery (granulocyte count > 1500/mm^3^, platelets count > 100.000/mm^3^) from the prior course of chemotherapy. After 1 week of delay, if the granulocyte count was between 1000/mm^3^ and 1500/mm^3^ and/or platelets were between 75.000/mm^3^ and 100.000/mm^3^, the doses of all drugs were reduced by 50%.

Sequential chest irradiation (60 Gy in 30 fractions) was also administered to patients with LD achieving CR or partial response (PR).

In patient with LD who achieved complete response (CR) or partial response (PR) prophylactic cranial irradiation (PCI) was administered; the radiation dose to the whole brain was 2.5 Gy in 10 fractions.

In selected cases of ED patients the whole brain radiotherapy (2.5 Gy in 10 fractions) was allowed according to clinical evaluation.

### Response and toxicity evaluation

Clinical response was evaluated according to RECIST criteria (version 1.0).

Clinical response was evaluated by repeating CT scan and all previously abnormal tests. after three and six cycles.

Toxicity, based on CTCAE toxicity criteria (version 3.0), was evaluated weekly and at the end of treatment.

Two patients that received only 1 course of chemotherapy were not considered evaluable for response.

### Statistical analysis

Overall survival (OS) time was measured from the date of diagnosis until death.

Survival time of patients who did not experienced the event considered during follow up observation was censored at the time of the last follow-up.

OS analysis was performed in all the 46 patients.

Time to progression (TTP) was calculated from the start of treatment to the date of first disease progression or relapse.

TTP of patients who did not experienced the event considered during follow up period was censored at the time of the last follow-up or at death, if occurred.

TTP analysis was performed in all the 44 patients considered evaluable for response.

Main statistical analysis has included: descriptive statistics of the main patients characteristics (gender, age, stage, performance status, metastatic site), estimation (with Kaplan-Meier method) and plot of survival probabilities for OS and TTP (stratified according to stage).

We used the statistical packages R 3.1.0, SAS 9.2 and PASW Statistics 18.0 (SPSS, Chicago, IL) for data analysis and visualization.

Dose intensity (DI is the drug dose delivered per time unit and is expressed as mg/m2 per week) was calculated for each drug of PEI regimen.

## Results

Between December 1998 and December 2008, 46 consecutive SCLC patients were treated in first line of chemotherapy with PEI regimen. The study population was composed of 30 male (65%) and 16 female (35%) with a median age of 63 years (range 59–70) (Table 1).

**Table 1:** **Study population: 46 patients treated with PEI**

	**N° Patients**	**(%)**
**Sex**		
Male	30	65
Female	16	35
**Age** (Yrs)		
Median	63	
Range	59-70	
**Stage**		
LD	19	41
ED	27	59
**PS (WHO)**		
0	29	63
1	13	28
2	4	9

Twenty-seven patients (59%) had ED and 19 (41%) had LD; the PS, graded according to the World Health Organization (WHO), was 0 in 29 cases (63%); 1 in 13 (28%) and 2 in 4 patients (9%). Metastatic sites were: lung (85% of the patients), mediastinal node (80%), liver (28%) and bone 24%. 8 patients had brain metastases at diagnosis.

There was no substantial significant difference in metastatic sites between genders: on average, in females group there were a mean of 2.9 metastatic sites, compared to 2.7 in the males (p-value 0.643, two tailed Poisson test).

Patients received a total of 219 cycles of chemotherapy, with a mean of 4,7 cycles per patient.

Dose Intensity (DI) was calculated for each drug of PEI regimen. Based on DI, the patients were divided in two groups, if they received < 80% or ≥80%. The median DI of cisplatin was 88%; Ifosfamide was administred with a median DI of 89% and the median DI of etoposide was 91%.

### Response and survival

In 19 LD patients an objective response (OR), defined as complete response (CR) or partial response (PR), was observed in 17 patients (90%), with 74% of PR and 16% of CR.

In 27 ED patients an OR was observed in 24 cases (89%), with 63% of PR and 26% of CR (Table 2).

**Table 2:** **Response (46 Pts): limited and extended disease**

**Limited Disease**	**NV**	**PD No (%)**	**CR No (%)**	**PR No (%)**	**SD No (%)**
	0 (0)	0 (0)	3 (16)	14 (74)	2 (10)
**Extended Disease**	**NV**	**PD No (%)**	**CR No (%)**	**PR No (%)**	**SD No (%)**
	2(7)	1 (4)	7 (26)	17 (63)	0 (0)

43 patients have died, 27/27 in ED (100%), 16/19 in LD (84%).

**Figure 1:**
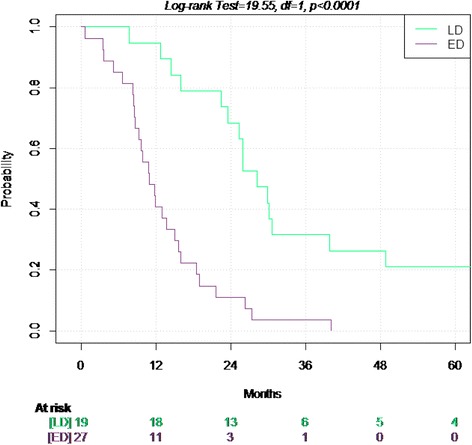
Overall Survival in limited and extended disease.

**Figure 2:**
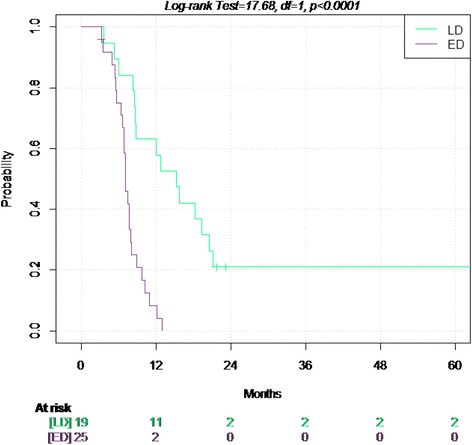
Time to progression in the limited and extended disease.

Figures 1 and 2 show the survival probabilities for OS and TTP up to the fifth year of follow up, stratified according to stage.

In the 46 patients, the median Time to Progression was 8.2 months (95%. CI 7.1-12): 15.2 months in LD and 7.1 months in ED.

The median OS in the 46 patients was 16 months (95% CI 13–25.9): 28.2 months in LD and 11.8 months in ED patients.

### Toxicity

Despite the use of antiemetic treatment, nausea was very common on the days of cisplatin infusion; however vomiting occurred only in 14% of patients.

Myelosuppression was the most frequent adverse event, with G3-4 neutropenia in 56%, anemia in 27% and thrombocytopenia in 21% of the patients.

27% of patients had febrile neutropenia.

Three toxic deaths were observed, all in patients with PS ≥ 1: two patients with sepsis and febrile neutropenia and one for heart failure in a cardiopatic patient.

Nonhematologic toxicity consisted mainly of asthenia (27%), mucositis (14%), nausea and vomiting (14%) (Table 3).

**Table 3:** **Grade 3 and 4 toxicities**

	**(%)**
**Leukopenia**	65
**Granulocytopenia**	56
**Anemia**	27
**Thrombocytopenia**	21
**Febrile neutropenia**	27
**Asthenia**	27
**Mucositis**	14
**Vomiting/Nausea**	14
**Diarrhea**	2
**Alopecia**	2
**Neurotoxicity**	2
**Anorexia**	0
**Venous thrombosis**	12
**Pulmonary embolism**	8
**Heart failure**	2

## Discussion

Small cell lung carcinoma (SCLC) which constitutes about 15% of all lung cancer, is a very aggressive tumor with a tendency for early metastasis [1].

Targeted therapy agents are widely used in NSCLC, but no one is approved for use in SCLC; in fact all the clinical trials investigating biological agents in SCLC, yielded negative results.

Imatinib did not demonstrate clinical efficacy even in patients with c-kit expression [13].

Vandetanib failed to demonstrate efficacy as a maintenance therapy and cediranib failed to demonstrate objective responses in recurrent or refractory SCLC [14].

Despite its peculiar chemo and radio-sensitivity, long term survival is achieved in a small minority of patients and combination of cisplatin and etoposide (PE) still represent the standard of care after 25 years; with this treatment objective response is achieved in 50-70% of patients but median survival of 7–8 months in ED [7].

Two randomized phase 3 trials evaluated doxorubicin-based chemotherapy compared with platinum-based chemotherapy in SCLC. Baka et al. enrolled patients with either LD or ED- SCLC who were randomized to doxorubicin, cyclophosphamide and etoposide (ACE) or PE for six cycles. There were no differences in response rates or in median survival time for LD (10.9 vs 12.6 months) and ED (8.3 vs 7.5 months) [15].

A Cochrane systemic review of platinum vs non-platinum chemotherapy regimens evaluated 29 trials with 5530 patients and concluded that platinum-based chemotherapy regimens did not offer a statistically significant benefit in either overall response or survival compared to non-platinum chemotherapy.

Subsequentely, a Japanase study, in 2002, reported that irinotecan and cisplatin combination resulted in a substantial improvement in survival compared with treatment with PE (median survival 12.8 vs 9.4 months) in ED [16].

The authors concludes that the combination of irinoteca and cisplatin was an attractive option for patients with metastatic small-cell lung cancer who have a good performance status.

Numerous other drugs have been studied in combination with platinum, without substantial improvemet in median survival or response rate.

Combination chemotherapy of belotecan (is a new camptothecin-derivative antitumor agent that belongs to the topoisomerase inhibitors) and cisplatin showed promising efficacy comparable to currently available standard regimen with favorable non-hematologic toxicity profile [17].

However, its high hematologic toxicity profile seems to preclude general acceptance of this regimen as option for treatment of SCLC. We are wainting for the result of the phase III COMBAT study which is comparing the efficacy of belotcan plus cisplatin to etoposide plus cisplatin in patients with ED SCLC.

Recent data with newer agents as thalidomide and bevacizumab, allow some cautious optimism for future advances in the treatment of this complex and challenging disease.

Strategies that have evaluated an increase of total dose, of dose intensity, number of courses, or number of drugs, or alternation of non-cross-resistant drugs, have been unsuccessful. These approaches are not recommended outside clinical trials [18].

Some evidence suggests that adding thoracic radiotherapy to chemotherapy improves survival in patients with extensive-stage SCLC who have a complete response outside the thorax and at least a partial response within the thorax after three cycles of etoposide and cisplatin [19].

Immediate whole-brain radiotherapy is indicated in patients with brain metastases and intracranial hypertension or other neurological emergencies. In some series of patients with SCLC or NSCLC and brain metastases, whole brain radiotherapy combined with different chemotherapy regimens seemed to increase the risk of neurological toxic effects, but also to increase response rates and lengthen the time to progression of brain metastasis [20,21].

This increase in toxic effects was probably related to the use of anthracyclines and high doses of radiation per fraction. On the basis of this evidence whole-brain radiotherapy should be started after the completion of chemotherapy in patients with brain metastases, with or without symptoms, but not delivered concomitantly with cytotoxic treatment.

Loehrer PJ et al., compared PEI regimen (cisplatin-etoposide-ifosfamide) with PE regimen in previously untreated ED-SCLC. The results indicated that PEI regimen was associated with a significant improvement in OS and PFS, without significant difference in toxicity [10–12].

The Hoosier Oncology Group study showed an improved time to progression (statistically different) and overall survival associated with PEI combination chemotherapy (9.0 month versus 7.3 with PE regimen), in 171 patients randomized from 1989 and 1993; in the previous study, reported in 40 patients from 1987 to 1989, PEI regimen produced a high complete remission rate in patients with extensive disease.

Ifosfamide combination chemotherapy demonstrated a durable complete remissions also in heavily pretreated patients with recurrent germ cell tumors [22].

In our study, PEI regimen gave interesting results both in LD and ED SCLC, with a remarkable median overall survival of 28.2 months in LD, and of 11.8 months in ED.

In both stages, the OS and TTP appear higher than expected, comparing to the results obtained with PE regimen published in the literature.

## Conclusions

SCLC remains a disease with disappointing results and no good news coming from the research. In extended-stage disease new drug combinations and approaches did not result in any improvement in overall survival, that remains the ultimate goal as, unlike in other chemosensitive cancers, second-line treatment is not an option for most patients.

Many other strategies, including maintenance therapy, dose-intense, dose-dense chemotherapy and alternating regimens have failed to demonstrate consistent benefits, often with unacceptable toxicity.

In the absence of a firm evidence of any activity of biological agents, PEI regimen, in our opinion, may be a possible therapeutic option, with high activity and an acceptable toxicity profile.
